# Fabrication of Hydrophilic Surface on Rigid Gas Permeable Contact Lenses to Enhance the Wettability Using Ultraviolet Laser System

**DOI:** 10.3390/mi10060394

**Published:** 2019-06-13

**Authors:** Hsin-Yi Tsai, Yu-Chen Hsieh, Yu-Hsuan Lin, Han-Chao Chang, Yu-Hsiang Tang, Kuo-Cheng Huang

**Affiliations:** Taiwan Instrument Research Institute, National Applied Research Laboratories, Hsinchu 30076, Taiwan; kellytsai@narlabs.org.tw (H.-Y.T.); yuchen820606@narlabs.org.tw (Y.-C.H.); marklin@narlabs.org.tw (Y.-H.L.); roman@itrc.narl.org.tw (H.-C.C.); sky520830@narlabs.org.tw (Y.-H.T.)

**Keywords:** rigid gas permeable contact lenses, wettability, hydrophilic, hydrophobic, 355 nm UV laser, surface treatment, microstructure

## Abstract

The widely used rigid gas permeable (RGP) contact lenses provide higher oxygen permeability and tear exchange rate than do soft contact lenses. However, their wettability warrants improvement to enhance the wearing comfort. This study used UV laser (wavelength = 355 nm) to modify the surface properties of RGP contact lenses with materials of Boston XO^®^ (Bausch & Lomb Incorporated). Briefly, the mesh pattern was fabricated on the RGP contact lens surface by using the laser and smoothed by using oxygen plasma; the enhanced hydrophilic efficiency was analyzed using contact angle measurement. The experiment results indicated that the contact angle of the lens material decreased by approximately 10°–20° when the pitch of mesh pattern was <50 μm under a 500-mm/s scanning speed. The oxygen plasma enhanced surface wettability with a decreased contact angle (40°). The hydrophilic characteristic of the UV laser and oxygen plasma–treated surface was twice that of oxygen plasma–treated and untreated surfaces. In the future, RGP contact lens edges could be treated with UV laser and oxygen plasma to enhance the tear wettability and wearing comfort.

## 1. Introduction

Contact lenses are divided into rigid gas permeable (RGP) and soft contact lenses according to the hardness of the lens material. Silicone hydrogel and poly(methyl methacrylate) (PMMA) are commonly used as main material for soft and hard contact lenses, respectively. Two critical characteristics determine the quality of both soft and RGP contact lenses: Permeability [1,2] and wettability [3,4]. Soft contact lenses are typically manufactured using water-containing, gel-like plastic materials, which are pliable and comfortable to wear. However, after several hours of wearing these lenses, the water gradually evaporates, impeding oxygen permeation through the lens. Consequently, as the cornea becomes hypoxic, the wearer’s eyes begin feeling dry. Compared with general soft contact lenses, RGP contact lenses provide high oxygen permeability and tear exchange rate [5]. For improving their permeability, high oxygen permeable materials such as silicone and fluorine are added to soft and RGP contact lenses, respectively. These materials enable rapid permeation of oxygen toward the cornea. This increases the lenses’ wettability to prevent microbial infections [6]. In addition, surface treatment and material modification are commonly used in industries and laboratories to increase the wettability of contact lenses.

Wettability is one of the most crucial properties determining a material’s solid surface and material applications [7]. In recent years, numerous studies have reported on surface hydrophilicity and hydrophobicity [8]. Several methods, such as chemical treatment, mechanical and flame method, ion-assisted reaction, and electron beam irradiation, can be used to transform normal material surfaces into hydrophilic or hydrophobic status. A well-designed microstructure can also be used to control surface roughness and alter its wettability [9,10]. The contact angle of the droplet on the surface can be enhanced to >160°. The most common example of this phenomenon is the lotus-leaf effect; in that, the numerous microstructures present on a lotus leaf render it superhydrophobic, with self-cleaning properties. Laser processing is another common method used in industries to change the surface roughness and wettability of material. By manufacturing different types and depth patterns on material surfaces, we can obtain different levels of material surface wettability. In a study, a femtosecond laser was employed to fabricate the micro or nano pattern on the polytetrafluoroethylene substrate [11]. The process parameters, such as the scanning speed, fluence, and beam overlap were adjusted, and uniaxial and biaxial patterns were produced to investigate the wettability of the patterns [11]. The results revealed that the biaxially scanned samples had superhydrophobicity properties and exhibited high contact angles and low contact angle hysteresis. Cai et al. [12] suggested that the dimensionless functional parameter R_hy_ and I_s_ are the most sensitive to the water contact angle of the specimen. Herein, the R_hy_ was the average ratio of the maximum height of the profile to the mean width of the profile element, and I_s_ was the average laser pulse energy per unit area of the specimen. Moreover, the R_hy_ and I_s_ of 0.41 and 536 J/mm^2^, respectively, can aid in ensuring the superhydrophobicity of the specimen during laser ablation. In addition, laser treatment on the surface of the substrate can be used to reduce the contact angle and produce a hydrophilic surface [13,14]. Lawrence et al. [15] used CO_2_, Nd:YAG, excimer, and high-power diode laser (HPDL) radiation to treat the surface of the biomaterial PMMA, and their results revealed that the variation in wettability of CO_2_-, Nd:YAG-, and HPDL-treated PMMA surfaces was small; by contrast, the wettability of the excimer laser radiation–treated PMMA surface considerably improved.

In addition, the effect of functional terminal groups with regard to wettability is widely known [16]. For instance, the terminal trifluoromethyl group (CF_3_) leads to a high contact angle situation, which represents the hydrophobic property of material surfaces. By contrast, some functional terminal groups, such as the hydroxyl group (SiOH), result in low contact angle and hydrophilicity of a material surface. Bodas et al. [17] used the conventional (oxygen plasma), unconventional plasma modification (oxygen and C_2_F_6_), and the oxygen plasma polymerization of 2-hydroxyethyl methacrylate (HEMA) process to modify the surface properties of poly(dimethyl siloxane) (PDMS). The results indicated that the surface with two days of hydrophilic stability was modified by oxygen plasma, and the surface with one week of good hydrophilic stability was modified by oxygen and C_2_F_6_. In addition, the chemical modification using HEMA provided the most efficient hydrophilic stability over 10 days. Plasma surface treatment is commonly used to form hydrophobic and hydrophilic surfaces [18]. Du et al. [19] coated the polytetrafluoroethylene (PTFE) on high–aspect ratio nanostructures and obtained hydrophobic surfaces. They employed the oxygen plasma etching and laser interference lithography to create hierarchical nanostructures, which resulted in superhydrophobic surfaces. With the aforementioned treatments, the droplet mobility on the surface of a slippery nanostructure was also enhanced by the hierarchical nanostructures. Kim et al. [20] fabricated nanospikes on a polyimide surface through plasma etching and fabricated hydrophilic microgrooves on the hydrophobic nanospike surface through laser ablation. In addition, colloidal silver was dipped in and attached on hydrophilic microgrooves. Thus, the zone affected by the laser heat should be reduced to reduce the line width of hydrophilic patterns, and these hydrophilic patterns of the hydrophobic surface can be used for cell growth, protein manipulation, and microfluidic collection.

For contact lenses, wettability also indicates how easily liquids spread over the lens surface; this can be observed through the interaction between the lens surface and tears and be defined by the contact angle [21]. The sessile drop, captive bubble, and Wilhelmy plate methods can be used to measure the contact angle and determine the wettability of a contact lens. Based on the aforementioned methods, measuring the contact angle of a contact lens without the ISO standard is difficult. Therefore, the standardized process of each technique should be built to reduce the measurement variations in contact angles. Cheng et al. [22] used the captive-bubble technique to measure the advancing and receding contact angles of two commercial silicone hydrogel lenses and reported that all the lenses had a considerable contact angle hysteresis with an advancing angle of almost 90° in the isotonic solution. In addition, when the lysozyme or mucin were added in the solution, the advancing and receding contact angles decreased because of the molecular absorption of proteins on the lens and indicated that the high water wettability of a lens can eliminate the protein absorption. Lin et al. [23] used the pendant-drop technique to measure the air and aqueous surface tension as well as the contact angle of soft contact lenses removed from the blister packs. The influence of the surface-active agents (surfactants) was also analyzed. The results revealed that the surface tension of all blister pack solutions was lower than that of pure water. Moreover, the wettability of most lenses was determined by the surfactants; the corresponding value decreased with the depletion of surfactants. By contrast, the SiH lenses exhibited a stable and self-sustained surface wettability.

No previous study has focused on enhancing the wettability of RGP contact lenses by fabricating patterns using laser technology. Herein, laser technology offered advantages such as rapidity and pattern flexibility for the Boston XO^®^ (Bausch & Lomb Incorporated, Rochester, NY, USA) RGP contact lens. On exposure to CO_2_ and Nd:YAG lasers, Boston XO^®^ RGP contact lenses absorb energy and melt because of the ablation mechanism which, at this wavelength, is mainly driven by a thermal effect in the microstructure fabrication process. Therefore, we employed an ultraviolet (UV) laser system to modify the surface properties of XO RGP contact lenses. The potential volume of the droplet was selected through patterned and unpatterned RGP contact lens surfaces. In addition, the laser system’s processing parameters, such as scanning speed and line pitch of the ablated pattern, were adjusted, and its effect on the surface roughness of RGP contact lenses and contact angle of the droplet were analyzed simultaneously. Therefore, the suitable parameters in the laser ablation process to fabricate a hydrophilic surface can be determined. Based on the experimental results, the hydrophilic surface on the material of RGP contact lenses can be fabricated through laser ablation. This process can aid in enhancing tear exchange intervals and opportunities when the lenses are worn. This method provides fast results, customized patterns, and wearing comfort.

## 2. Fundamental Theory

A tear has a high adhesive force and thus can cause droplets to spread on the surface of the eyes and the surrounding tissue. Although soft contact lenses are made of high water content material, the water content in RGP contact lenses is relatively low, and the hydrophilicity between the RGP contact lens and eyeball surface needed to be improved to ensure that the eyes will not be dehydrated. The contact angle (*θ*) represents the angle at the liquid–vapor and solid–liquid interfaces [24,25]. In general, a contact angle of <90° indicates that the solid material has a wettable surface and thus is highly hydrophilic, whereas if the contact angle is >150°, the solid material is highly hydrophobic. In highly hydrophobic materials, liquids cannot enter the microstructure of the solid surface and thus the interface between the solid material and the liquid becomes small. This phenomenon is called the lotus-leaf effect. 

Because the RGP contact lens surface is not perfect, contact angle hysteresis occurs from the difference between the advancing and receding contact angles (*θ_A_* and *θ_R_*). In addition, the equilibrium contact angle (*θ_e_*) can be described as follows [26,27,28,29]:

(1) $$\theta_{e}=\cos^{-1}\left(\frac{\gamma_{A\;}\cos(\theta_{A})+\gamma_{R}\;\cos(\theta_{R})}{\gamma_{A}+\gamma_{R}}\right)$$

where

(2) $$\gamma_{A}={\left(\frac{\sin^{3}(\theta_{A})}{2-3\cos\left(\theta_{A}\right)+\cos^{3}\left(\theta_{A}\right)}\right)}^{\frac{1}{3}}$$

(3) $$\gamma_{R}={\left(\frac{\sin^{3}(\theta_{R})}{2-3\cos\left(\theta_{R}\right)+\cos^{3}\left(\theta_{R}\right)}\right)}^{\frac{1}{3}}$$

**Figure 1:**
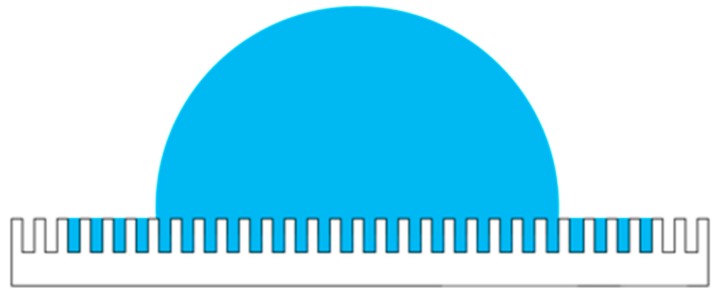
Droplet on microstructures according to the Wenzel model.

Rough surfaces increase hydrophilicity of the contact lens; these rough surface textures can be divided into homogeneous and heterogeneous textures. The current study aims to reduce the contact angle of a droplet on the RGP contact lens by modifying the surface of the RGP contact lens by using a laser system and increase its hydrophilicity. The classic descriptive theories of the contact angle of a droplet on microstructure surfaces include the Wenzel and Cassie–Baxter models [30,31,32,33]. The contact angle hysteresis can be ignored because the contact lens is static, and the droplet would not flow when worn. Therefore, the surface of the material treated with the laser system was maintained as a homogeneous surface, and the phenomenon can be described by the Wenzel model, as illustrated in Figure 1 and as written in Equation (4),

(4) $$\cos\left(\theta^{*}\right)=r\;\cos\left(\theta \right)$$

where θ* is the static contact angle and r is the roughness ratio of the material surface, which is the ratio of the true area of the object to the apparent area. In addition, *θ* is the Young contact angle, which represents the contact angle of the ideal surface.

When the surface of the material has microstructures, a higher hydrophilicity can be achieved when the surface roughness is finer. The microstructures on the contact lenses provide spreading and imbibition abilities, and the surface is defined as the highest hydrophilicity with a contact angle less than π/2 [34]. If it is lower than the critical contact angle of the model, there will be a formation of liquid film on the surface, which is called ultrahigh hydrophilicity.

## 3. Materials and Experimental Setup

### 3.1. Materials

PMMA is a stable material. Its mechanical or optical properties are not significantly affected when it is exposed to a high-humidity environment or is infiltrated with water. Herein, the contact angle of the flat PMMA was 77.5° when the volume is 3 μL. Here, the RGP contact lens material XO was purchased from Bausch & Lomb Incorporated. In addition to the conventional PMMA material, silicon and fluorine were added to the XO to allow more oxygen to pass through the lens and reach the patient’s eye, and the material was defined as the hexafocon A. The Boston XO^®^ RGP contact lenses have an ultrasmooth surface and are nonstick; they thus resist dirt and debris. The customized design of the lens provides a perfect fit and natural shape for the patient’s eyes. The wetting angle of XO is 49°, and its oxygen permeability is 100 ((cm^3^ [O_2_]·cm)/(cm^2^·s·mmHg)) [35].

### 3.2. Experimental Setup

#### 3.2.1. Surface Treatment: UV Laser (355 nm) Processing and Oxygen Plasma Cleaning

**Figure 2:**
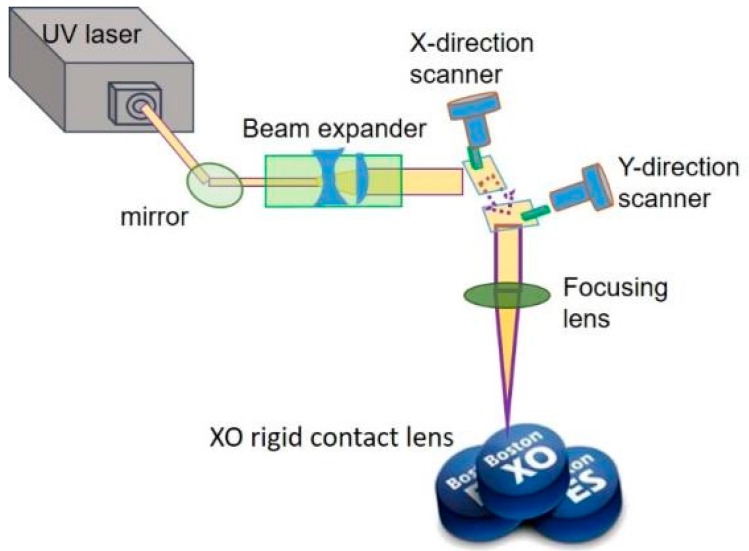
Laser treatment system setup on Boston XO^®^ rigid gas permeable (RGP) contact lens.

The laser source was a diode-pumped solid-state UV laser (Coherent, Inc. AVIA 355-14^TM^, Santa Clara, CA, USA) with a wavelength of 355 nm and a maximum average output power of 14 W, operating a pulse repetition frequency range of 1–400 kHz. Its each pulse width is of 32 ns at 40-kHz pulse repetition rate, transverse mode is TEM^00^ with an output beam diameter of 3.5 mm, and beam quality factor (e.g., M^2^) is <1.3. Here, its beam diameter was magnified by a 2× beam expander. High speed galvanometric scanning mirrors were used to transform the direction of the laser beam for laser processing and improve processing efficiency (Figure 2). A telecentric focusing lens module with a 160-mm focal length was used; the theoretical and actual diameters of the focused laser spot on the working plane were approximately 13.5 and approximately 30 μm, respectively.

**Figure 3:**
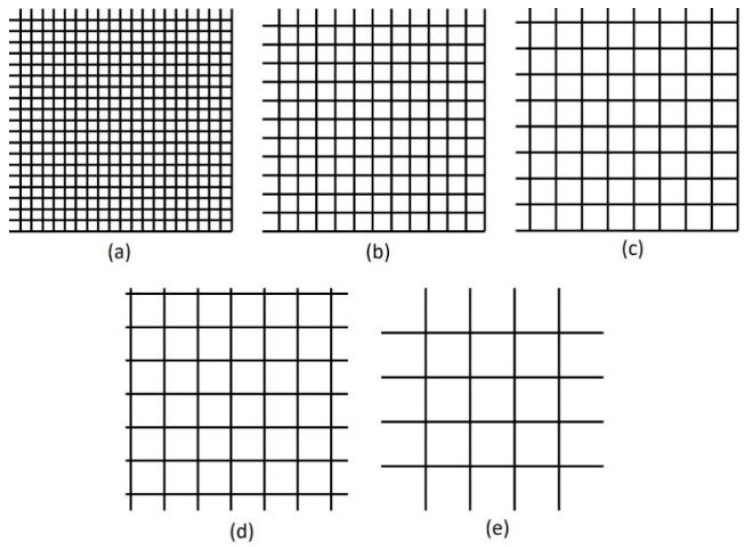
Mesh patterns with pitch of (**a**) 30, (**b**) 50, (**c**) 70, (**d**) 90, and (**e**) 110 μm.

First, we used the UV laser to make patterns on the surface of the material of the RGP contact lens. The scanning speed of the laser spot and spacing of the laser scanning were adjusted and designed to make mesh patterns with various depths, line pitch, and roughness. The output power of the laser system was set at 10 W, and the pulse repetition frequency was fixed at 100 kHz. Laser scanning speeds of 500, 1000, 1500 and 2000 mm/s were used. The line pitch of the mesh pattern ranged from 30 to 110 µm at 20-µm intervals. The mesh patterns with five line pitches are illustrated in Figure 3. After the surface treatment with the UV laser, an oxygen plasma cleaner (HARRICK PLASMA, PLASMA CLEANER PDC-32G, Ithaca, NY, USA) was employed to clean the splash on the surface material caused by the laser processing and modify the surface properties of the material of the RGP contact lens. For the oxygen plasma cleaning parameters, the input power was 100 W, the power applied to the RF coil was 11 W, and the cleaning lasted for 10 min.

#### 3.2.2. Contact Angle Measurement and Surface Morphology Observation

After the surface treatment of the lens with the laser, the treated lens’s surface morphology and roughness were observed and measured using a three-dimensional confocal laser scanning microscope (KEYENCE, VK-X200, Itasca, IL, USA) and its analysis software program (KEYENCE, VK-Analyzer Plus™, Itasca, IL, USA). In addition, the contact angle of the droplet on the Boston XO^®^ RGP contact lens related to its wettability was measured through contact angle measurement (FTA 188, First Ten Angstroms Inc, Portsmouth, VA, USA).

#### 3.2.3. Experimental Process

Four primary steps were conducted to obtain the contact angle and determine the performance of the treated pattern on the XO RGP contact lens surface; the adjusted and measured target of each step are described in detail as follows:

Step (I): The AutoCAD package was used to design the ablation path of the laser spot. The parameter of the pitch of two ablated lines was adjusted to range from 30 to 110 μm with intervals of 20 μm.
Step (II): The parameters, such as the power and pulse repetition frequency of the laser system, were fixed, and the scanning speed of the laser spot was adjusted to generate various spot overlaps, line widths, and surface roughness.
Step (III): A 3D confocal microscope was used to measure the morphology of the ablated mesh pattern and analyze the relationship of the real line pitch, width, and surface roughness with the scanning speed during laser treatment. Herein, the surface roughness was the mean height (Ra).
Step (IV): The XO RGP contact lens was placed into an ultrasonic oscillator with DI (de-ionized) water for 10 min to clean its surface, and nitrogen (N_2_) was used to blow dry its surface. The lens was then processed for contact angle measurement, for which a 3-μL droplet of physiological saline was added to the lens. The contact angle was measured. In addition, the relationship between the contact angle and surface roughness of the XO RGP contact lens was analyzed to obtain the best pattern to reduce the contact angle and enhance the wettability of RGP contact lenses.

## 4. Experimental Results and Discussion

To investigate the wettability of the XO RGP contact lens, affected by the volume of a droplet and the surface properties, after the laser treatment, the contact angle and surface roughness of each lens was analyzed. The measured contact angle was the average of the eight values measured from four directions in a circle at the intervals of 90°. The measurement was repeated twice. Surface roughness was the average value in the full of view of the measured area, which was approximately 1.6 × 1.0 mm^2^ under the 10× objective lens.

### 4.1. Analysis of the Contact Angle Affected by the Droplet Volume

Different volumes of physiological saline droplets have different gravity; therefore, the selectivity of the droplet volume was determined in the subsequent experiments. On the original surface of the XO RGP contact lens, the contact angle of the droplet was only affected by the gravity, and the increase in the droplet volume required a larger area and a higher base width to support the droplet. Therefore, the support of the droplet edge on the material’s surface caused the contact angle of the droplet to decrease simultaneously (Table 1). In addition, the mesh pattern with line pitches of 30 μm, treated with a laser scanning speed of 1000 mm/s, was fabricated on the lens surface. With the microstructures, the contact angle of the droplet is affected by the gravity, surface roughness, and pattern’s height. In this situation, the droplet infiltrated into the valley of microstructures, and the base width of the droplet increased only slightly with the increase in droplet volume, as summarized in Table 2. Therefore, sufficient surface area was generated to support the droplet and caused a similar contact angle when the droplet volume ranged from 3 to 5 μL (Table 1). Based on the aforementioned results, a droplet volume of 2 μL was too small to be affected by the surface morphology and a volume of 4–5 μL was too large and easily affected by the gravity and surface morphology. Moreover, a droplet volume of 3 μL started to be affected by the microstructures, indicating that this volume of droplet was significantly affected by the surface properties. Therefore, the droplet volume of 3 μL was determined in the following experiments to investigate the contact angle affected by the surface morphology and roughness.

### 4.2. Analysis of the Surface Morphology and Roughness

**Figure 4:**
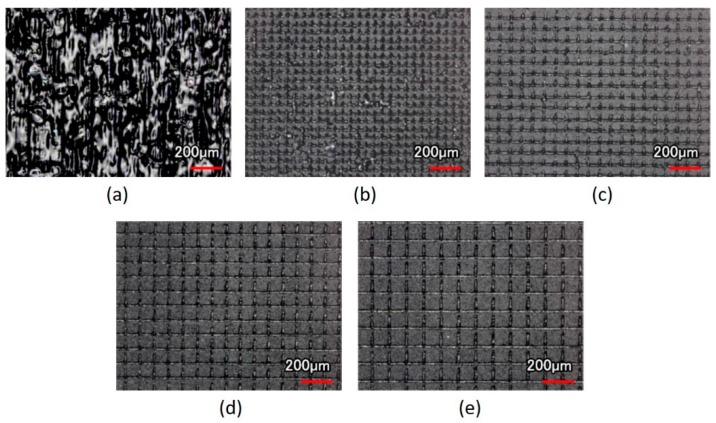
Optical images of the XO RGP contact lens treated with the laser system with a scanning speed of 500 mm/s and line pitches of (**a**) 30, (**b**) 50, (**c**) 70, (**d**) 90, and (**e**) 110 μm.

After the scanning speed of the laser spot was fixed and the scanning path of different line pitches was imported, the surface morphology of the contact lens treated with the laser system could be observed from the optical images (Figure 4). The size of the focused laser spot was approximately 30 μm, and the line pitches of 30 μm caused the laser spot on the XO RGP contact lens to close the next strip. Therefore, the full view of the laser scanning area was treated by the laser spot and caused melting and the rough surface and the largest surface roughness (Figure 4a).

**Figure 5:**
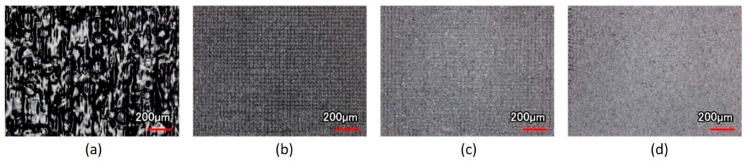
Optical images of the XO RGP contact lens treated with the laser system with line pitches of (**a**) 30 μm and a scanning speed of (**a**) 500, (**b**) 1000, (**c**) 1500, and (**d**) 2000 mm/s.

In addition, in the region where only the laser focused spot was ablated and the checkerboard microstructures were fabricated, the line pitches became wider, and the surface roughness would gradually decrease simultaneously, as summarized in Table 3. When the line pitch of the laser spot was fixed and the scanning speed was adjusted, the scanning speed affected the residence time of the laser spot; the treated depth was also altered (Table 4). The treated depth of the laser spot and the surface roughness decreased with the increase of the scanning speed (Figure 5), even without the ablation trace on the surface of the XO RGP contact lens (Figure 5d). The surface roughness and the root mean square of the height decreased from 13.002 to 2.230 μm and from 18.770 to 3.081 μm, respectively, when the scanning speed increased from 500 to 2000 mm/s. Herein, the roughness and root mean square height was defined by the average value and root mean square along the sampling length, respectively; there was no overlap in the laser trace when the line pitches of the microstructures were 90 and 110 μm. Thus, the variation of the surface roughness and root mean square height of mesh patterns in the aforementioned pitches were small when the scanning speed was changed. 

### 4.3. Analysis of the Contact Angle Affected by Line Pitch and Laser Scanning Speed

**Figure 6:**
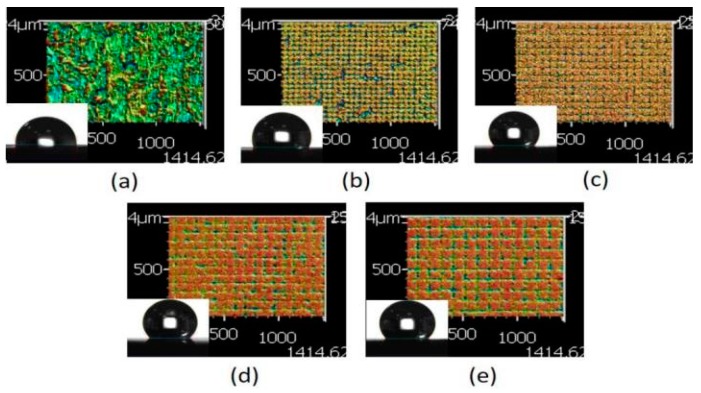
Three dimensional (3D) images of the XO RGP contact lens treated with the laser system with a scanning speed of 500 mm/s and line pitches of (**a**) 30, (**b**) 50, (**c**) 70, (**d**) 90, and (**e**) 110 μm.

When the physiological saline droplet was dropped on the surface of the treated RGP contact lens, the droplet infiltrated the melting surface and caused a lower contact angle (Figure 6a). The checkerboard pattern became clearer with the increase in the line pitch. Subsequently, the droplet was supported on the surface of the XO RGP contact lens, and the contact angle increased and reached 130° on the pattern with a line pitch of 90 μm. When the line pitch was 110 μm, the surface roughness increased again because of the more plate area and relatively higher position than the pattern with a line pitch of 90 μm in the same area. The droplet usually exists on the surface and infiltrates the valley of patterns at the droplet edge to cause the lowering of the contact angle of the droplet (Figure 6d,e).

**Figure 7:**
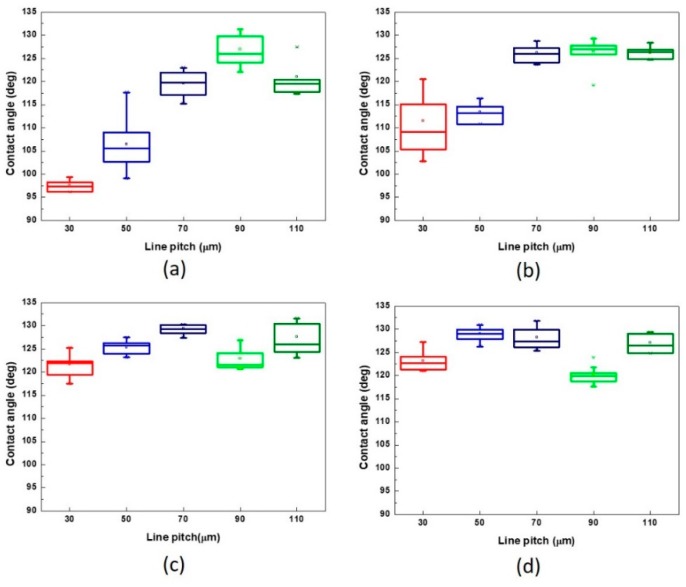
Contact angle of physiological saline droplet on the XO RGP contact lens with different line pitches under the scanning speed of (**a**) 500, (**b**) 1000, (**c**) 1500, and (**d**) 2000 mm/s.

When the pattern was fabricated using different scanning speeds and line pitches, the contact angle of the droplet on these patterns was measured; the results are presented in Figure 7. The results indicated that the contact angle of the droplet usually increased with the increase of the line pitch of the patterns treated by the same scanning speed, particularly in the range of 30–70 μm. It also represented that the contact angle of the droplet was evidently affected by the pitch of microstructures. When the line pitch of the pattern increased to 90 and 110 μm, there was sufficient platted region to support the droplet, and the contact angle would be affected by various factors such as the droplet position on the microstructures, surface roughness, and ablated depth of the pattern. Therein, the higher surface roughness was obtained from the pattern with the line pitch of 110 μm than 90 μm while treated by the laser system with a scanning speed of 500 mm/s, shown as Table 3 and Figure 6, and the droplet major exists on the platted region and may infiltrate into the valley of patterns when the line pitch of pattern increased. Thus, the contact angle of the droplet has a maximum value on the pattern with a line pitch of 90 μm. (Figure 7a) Therefore, smaller line pitches were more suitable to be selected to fabricate the microstructures and reduce the contact angle of the droplet. In addition, there was no laser trace on the XO RGP contact lens when the scanning speed was higher than 1500 mm/s. Therefore, the variation of the contact angle of the physiological saline droplet on the XO RGP contact lens was small between the scanning speed parameters of 1500 and 2000 mm/s, as illustrated in Figure 7c,d.

**Figure 8:**
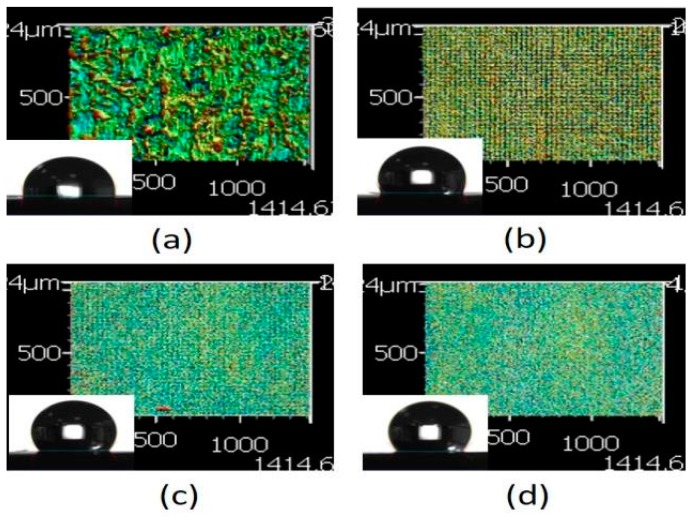
3D images of the XO RGP contact lens treated with the laser system with line pitches of (a) 30 μm and a scanning speed of (**a**) 500, (**b**) 1000, (**c**) 1500, and (**d**) 2000 mm/s.

Based on the aforementioned results, the pattern with a line pitch of 30 μm was selected to analyze the effect caused by the laser scanning speed. Compared with the 3D profile of the patterns and the measured contact angle of the droplet (Figure 8), it revealed that the droplet infiltrated the melted surface and had the lowest contact angle of approximately 97° with the largest surface roughness of 13.002 μm when the pattern was treated with a scanning speed of 500 mm/s. By contrast, the surface was relatively smooth and exhibited almost no ablated trace when the scanning speed was >1500 mm/s; it had the lowest surface roughness when the pattern was treated with a scanning speed of 2000 mm/s. Therefore, the contact angle of the droplet was usually >120° on the aforementioned patterns. It remains a hydrophobic surface under these treatment parameters, and the wettability was poor than the XO RGP contact lens without laser treatment.

**Figure 9:**
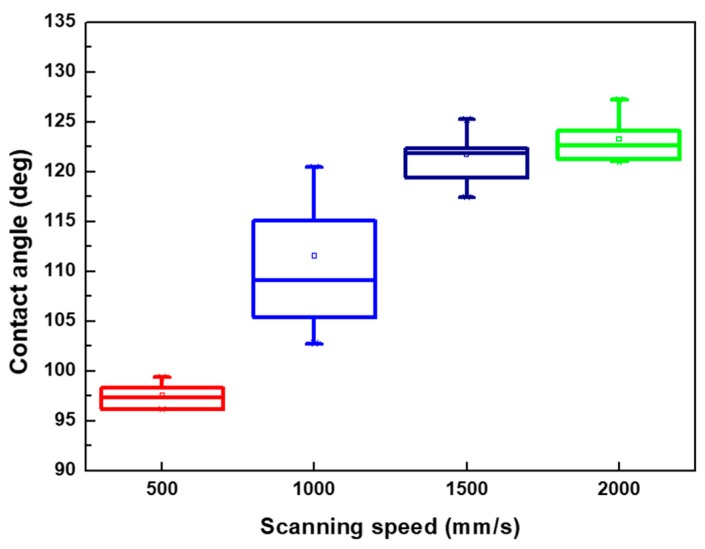
Contact angle of physiological saline droplet on the XO RGP contact lens with line pitch of 30 μm under four various laser scanning speeds.

**Figure 10:**
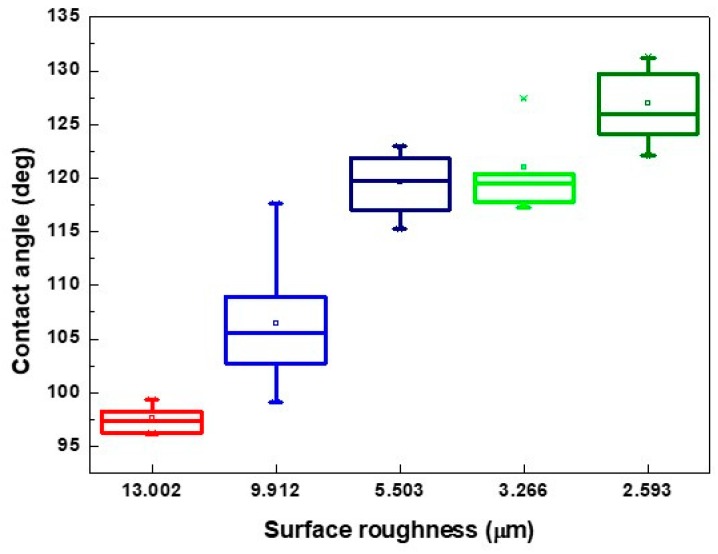
Contact angle of physiological saline droplet on the XO RGP contact lens with five surface roughness under laser scanning speed of 500 mm/s.

From the surface roughness summarized in Table 3 and the contact angle of the droplet presented in Figure 7, Figure 9 and Figure 10, the contact was usually >110° and even >120° when the surface roughness was <5 μm. When the surface roughness ranged from 5 to 10 μm, the contact angle could be reduced to 100°–122°. In addition, the surface of the XO RGP contact lens will gradually become hydrophilic when the surface roughness was enhanced to >10 μm, which was approximately in the range of 96°–99° when the surface roughness was approximately 13 μm. Therefore, a lower scanning speed of the laser system and a pattern with smaller line pitches should be employed to reduce the contact angle of the droplet and enhance its wettability.

### 4.4. Analysis of the Contact Angle Affected by Oxygen Plasma

Oxygen plasma cleaning is usually employed to remove surface impurities and contaminants by exciting gas atoms with high energy states and ionization. The OH- group will be introduced by the oxygen plasma and cause the decrease in the contact angle of the droplet on the surface. The lowest contact angle of the droplet was measured from the surface of the RGP contact lens with a line pitch of 30 μm UV treated with a laser with a scanning speed of 500 mm/s, which was approximately 20° lower than that on the smooth surface without UV laser treatment. To enhance the hydrophilic characteristics, the oxygen plasma was applied to the surface of the XO RGP contact lens, and the measured value of the contact angle was approximately 36° on both surfaces with and without UV laser treatment. However, the hydrophilic characteristics gradually decreased because the particle was attached on the surface and caused reduced hydrophilic properties when the XO RGP contact lens was used for several hours or days. Therefore, the surface of the RGP contact lens recovered to the hydrophobic surface, and the contact angle of the droplet increased to 101° after the 110 h of oxygen plasma treatment, as summarized in Table 5. By contrast, the surface of the RGP contact lens conserved its hydrophilic characteristics, and the contact angle of the droplet on the surface treated with UV laser treatment only increased to 55° after 110 h. It indicated that the surface of the RGP contact lens that was treated by UV laser and oxygen plasma had good hydrophilic characteristics even after four or five days, and its maintenance was better than the surface only treated with oxygen plasma.

## 5. Conclusions

To enhance the wettability of RGP contact lenses, a laser treatment was applied in this study to investigate the relationship between the contact angle and the line pitch of the pattern and its ablated trace and depth affected by the laser scanning speed. The results revealed that a lower contact angle and better wettability can be obtained when the surface roughness is increased by reducing the laser scanning speed or line pitches. The most hydrophilic surface was fabricated at a laser scanning speed of 500 mm/s and a line pitch of 30 μm, whereas the most hydrophobic surface was obtained when the surface roughness was <5 μm and fabricated from line pitches of >70 μm with all scanning speed and line pitches of 30–50 μm with a scanning speed of 1500–2000 mm/s. In addition, the oxygen plasma was employed to re-enhance the wettability after the laser treatment, and the contact angle decreased to 40° compared with that before the oxygen plasma treatment. The contact angle of the droplet on the surface treated with the UV laser and oxygen plasma treatment was half of that of the surface treated with oxygen plasma. In the future, the edge of the RGP contact lens can be treated to enhance the wettability of tears, and different patterns can be directly fabricated by the laser system. It has the advantage of being fast, not requiring a mask, and offering various pattern selectivities. Moreover, the pattern at the edge of the RGP contact lens would not affect the vision area.

## Figures and Tables

**Table 1:** Contact angle of the droplet on XO RGP contact lens with and without mesh patterns (Unit: °).

Pattern	Droplet Volume (μL)			
	2	3	4	5
Without mesh patterns	121 ± 6.1	118 ± 3.4	112 ± 3.5	104 ± 8.9
With patterns of line pitch 30 μm	118 ± 6.5	111 ± 8.7	114 ± 7.4	110 ± 6.2

**Table 2:** Base width of the droplet on XO RGP contact lens with and without mesh patterns (Unit: mm).

Pattern	Droplet Volume (μL)			
	2	3	4	5
Without mesh patterns	2.08 ± 0.14	2.35 ± 0.14	2.62 ± 0.10	2.98 ± 0.29
With patterns of line pitch 30 μm	2.14 ± 0.17	2.53 ± 0.13	2.55 ± 0.14	2.77 ± 0.15

**Table 3:** Surface roughness (Ra) of mesh patterns on the XO RGP contact lens treated by different laser scanning speeds and line pitches (Unit: μm).

Scanning Speed (mm/s)	Line Pitch (μm)				
	30	50	70	90	110
500	13.002	9.912	5.503	2.593	3.266
1000	6.032	2.698	2.642	2.797	2.507
1500	2.747	2.678	2.767	2.276	2.478
2000	2.230	2.885	2.506	2.781	2.754

**Table 4:** Root mean square height (Rq) of mesh patterns on the XO RGP contact lens treated by different laser scanning speeds and line pitches (Unit: μm).

Scanning Speed (mm/s)	Line Pitch (μm)				
	30	50	70	90	110
500	18.770	13.215	8.300	3.569	4.222
1000	7.989	3.689	3.573	3.752	3.391
1500	3.894	3.631	3.727	3.089	3.352
2000	3.081	3.856	3.409	3.762	3.682

**Table 5:** Comparison of contact angle of the droplet on the XO RGP contact lens treated by oxygen plasma (Unit: °).

Oxygen Plasma Treatment	Pattern	
	Without	Line Pitch of 30 μm
Before	118.03 ± 2.59	97.58 ± 1.29
after 1 h	36.22 ± 4.41	36.82 ± 2.28
after 110 h	101.18 ± 1.50	55.82 ± 3.13
